# Predicting progression to proliferative diabetic retinopathy using automated versus manual quantification of retinal haemorrhages

**DOI:** 10.1038/s41433-025-04205-2

**Published:** 2026-01-16

**Authors:** Aditya Verma, Muneeswar G. Nittala, Roxan Mansoori Dara, Marius Facktor, Chaithanya A. Ramachandra, Malavika Bhaskaranand, Sandeep Bhat, Kaushal Solanki, Chaitra Jayadev, Swetha B. Velaga, Gavin Robertson, Bradley Yates, Rajiv Raman, SriniVas R. Sadda

**Affiliations:** 1 Doheny Eye Institute, Pasadena, CA USA; 2 Department of Ophthalmology and Visual Sciences, University of Louisville, Louisville, KY USA; 3 School of Medicine, Meharry Medical College, Nashville, TN USA; 4 Eyenuk Inc, Los Angeles, CA USA; 5 Narayana Nethralaya, Bangalore, India; 6 Optos Research and Development, Optos plc, Dunfermline, UK; 7 Executive Vice President Product/Solutions Management, (EVP-Product) Optos, Dunfermline, UK; 8 Sankara Nethralaya, Chennai, India; 9 David Geffen School of Medicine, University of California, Los Angeles, Los Angeles, CA USA

**Keywords:** Retinal diseases, Predictive markers

## Abstract

**Purpose:**

To compare automated and manual quantification of retinal haemorrhages in eyes with diabetic retinopathy (DR) and to analyse the risk of progression to proliferative DR (PDR).

**Methods:**

Retinal haemorrhages on ultra-widefield (UWF) pseudocolor images in eyes with non-proliferative diabetic retinopathy (NPDR) were manually segmented. DR severity was assessed within the seven ETDRS fields at baseline and 1-year follow-up. Lesions were also automatically segmented using EyeRead UWF software (Eyenuk) and the frequency and area of retinal haemorrhages and the average distance of haemorrhages from the optic nerve centre were computed. Manual and automated results were compared and correlated with progression to PDR at one year.

**Results:**

Sixty-three eyes with NPDR at baseline were included, of which 29 progressed to PDR over one year. The automated measurements of total haemorrhage frequency, area and the distance from the optic nerve were significantly lower compared to manual grading, but the parameters were significantly correlated (r = 0.5–0.96; all *P* < 0.001). The distance of the haemorrhages from the optic nerve was found to be a significant risk factor for progression to proliferative DR from both approaches.

**Conclusions:**

Automated detection of retinal haemorrhages may serve as a surrogate to the manual grading in predicting the progression to PDR. Although the automated approach detects a lower number of lesions compared to manual grading, the results are correlated and the automatically determined parameters are still predictive of progression.

## Introduction

The staging systems for assessing the severity of diabetic retinopathy (DR) were developed decades ago in an era when 30–35° flash colour fundus photography was the widely available retinal imaging technology [1]. By steering the camera in various directions and capturing overlapping fields, larger regions of the retina could be surveyed, resulting in the establishment of the Early Treatment of Diabetic Retinopathy Study (ETDRS) 7 standard fields (7SF) [2]. Although the region covered by the ETDRS 7SF only spanned ~30° of the retina, this was deemed to be sufficiently representative of the overall impact of disease on the retina. Indeed, assessment of the severity of specific DR lesions, such as haemorrhages, microaneurysms, venous beading, intraretinal microvascular abnormalities (IRMA) and neovascularization within these fields compared to pre-defined standard photographs, allowed researchers and clinicians to arrive at a severity score or level of diabetic retinopathy in the eye [3]. This diabetic retinopathy severity score (DRSS) proved to be predictive of future progression of DR to more advanced stages, such as proliferative DR (PDR) in subsequent trials [2–4].

Over the last two decades, however, the ultra-widefield (UWF) pseudocolor imaging technique capable of imaging up to 90% of the retinal surface in a single capture has become broadly available [5–8]. Multiple studies have indicated a disconnect between the severity of diabetic retinopathy within the 7SF and the retinopathy evident in the more peripheral retina [7–9]. Subsequently, Silva and colleagues demonstrated that eyes with predominantly peripheral lesions (PPL), defined by the presence of at least one peripheral field having more extensive lesions than the corresponding central ETDRS field, had a dramatically higher risk for progression to PDR [10, 11]. It should be noted that the peripheral lesions identified by these researchers were primarily haemorrhages and microaneurysms. DRCR.net Protocol AA explored this question in greater detail and while only PPL determined by UWF fluorescein angiography (FA) were found to increase the risk for progression, the study did confirm the importance of evaluating the retina beyond the 7SF [12].

A limitation of Protocol AA and most studies and trials evaluating DR severity, however, is that they are still reliant on a subjective and categorical staging system that was developed in an era that was limited by small field analog film photography. In an attempt to develop a more objective approach to DR staging, we quantified the number and extent of all diabetic lesions to assess their severity numerically. We also measured the distance of each lesion relative to the optic nerve head (ONH) in order to define their eccentricity numerically. Using this approach, we were able to demonstrate that the total surface area of retinal haemorrhages and the mean distance of lesions to the ONH centre were independent predictors of the risk for progression from non-proliferative DR (NPDR) to PDR [13]. Interestingly, no other DR lesions (for e.g. IRMA) imposed independent additional risk to the model when considering the entire retina. This initial work was performed by tedious manual annotation of every lesion, in some cases 1000’s of lesions in a single eye, which is clearly impractical for broad utilisation in clinical or research practice.

Recently, advances in automated image analysis, specifically enabled by artificial intelligence, have allowed the possibility of detecting the retinal lesions automatically and quantified in an efficient and clinically feasible manner [14–28]. Although automated detection appears to fare better in detecting lesions in the central fundus when compared to the general ophthalmologist [29, 30], the accuracy rates of detection of lesions remain sub-optimal. Due to morphological variations of retinal haemorrhages, especially in the nerve fibre layer, the sensitivity rates of lesion detection/ annotation may vary with the algorithm used [18–20, 22, 25–28, 31, 32]. Furthermore, image artefacts in the peripheral fundus (e.g. lid margins and lashes) on UWF images can further impair the performance of these automated lesion detection algorithms [33–36]. Despite these challenges and even if they do not numerically match human annotations, it is still possible that automated DR lesion detection could correlate with other traditional DR staging approaches (e.g. modified ETDRS scale) and be predictive of DR progression.

This study investigates the feasibility of using a deep learning-based algorithm (EyeRead by Eyenuk, Inc.) [37] to quantify retinal haemorrhages from UWF images in eyes with NPDR. We compared these automated quantification metrics with manual expert annotations, focusing on their ability to predict progression to PDR. By addressing the challenges of manual grading and exploring the potential of automated detection, this study aims to contribute to the development of efficient, objective tools for DR management.

## Methods

### Study population

Consecutive patients diagnosed with non-proliferative diabetic retinopathy (NPDR) at the retina clinics at Narayana Nethralaya (Bangalore, India) and Sankara Nethralaya (Chennai, India) who had UWF pseudocolor imaging at baseline and at least one year of follow-up data were included in this retrospective study. All patients included in this analysis were seen between January and November 2017. Eyes with dense cataract or other media opacity precluding imaging, any myopic chorioretinopathy or axial length >24 mm or any other ocular diagnosis aside from NPDR were excluded from the analysis. The study adhered to the tenets of the Declaration of Helsinki and was approved by the Ethics Committee of Narayana Nethralaya, Bangalore, India (#15-000083) and the Ethics Committee of Sankara Nethralaya, Chennai, India (844-2017-P). Prior informed consent was obtained from all the subjects.

### Image acquisition

The UWF images were obtained at both centres after pupillary dilatation using a scanning laser ophthalmoscope, the Optos Daytona Plus (Optos plc, Dunfermline, Scotland, UK). Trained photographers captured two 200° central images and four steered peripheral images (superior, inferior, temporal and nasal quadrants). All images were transferred to the Doheny Image Reading and Research Lab (DIRRL) for further grading.

**Fig. 1:**
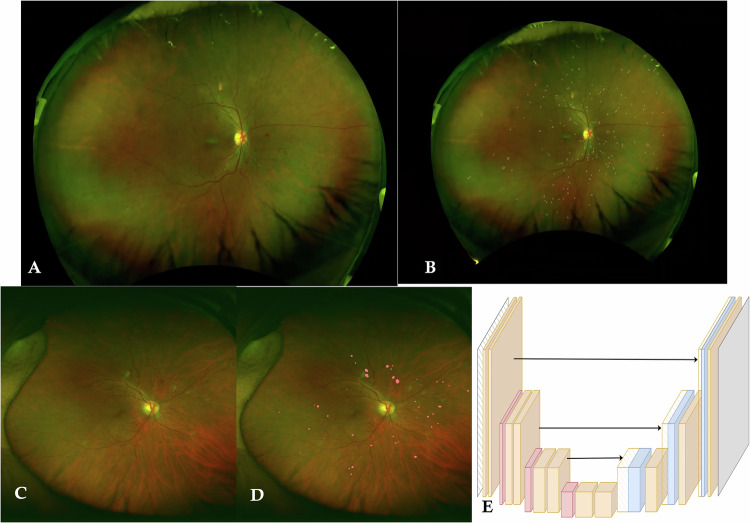
Manual and automated retinal haemorrhage segmentation. **A**, **B** show the ultra-wide field pseudo-colour fundus image of the right eye and the manually segmented retinal haemorrhages using the Grador software, respectively. **C**, **D** show the ultra-wide field pseudocolor fundus image of the right eye and the automatically segmented retinal haemorrhages using EyeRead software from Eyenuk Inc. **E** shows the schematic illustration of the neural network for localisation of haemorrhages on UWF image, using the AI software.

### Grading protocol (Fig. 1)

The graders utilised all images to determine the DR severity. However, only a single central image having the maximum gradable field of view at both baseline and at the 1 year-follow up visit was selected for quantitative lesion analysis. Stereographic projection was applied to correct peripheral distortions using the manufacturer’s software and to allow anatomically correct physical measurements to be generated with regard to lesion (retinal haemorrhages) area and distance of each lesion to the optic nerve centre. Manual segmentation of every retinal haemorrhage lesion in every baseline image was performed by two highly experienced senior reading centre graders (MGM and SBV). To accomplish this, the images were imported into custom-designed GRADOR software, which has been described in prior reports [38, 39]. Boundaries of all visible retinal haemorrhages were carefully segmented throughout all retinal areas visible in the UWF image (Fig. 1A, B). For reproducibility analysis, 20% of the baseline data were regraded by two independent graders (AV and RMD) at a later date. DRSS grading was performed based on the ICDR severity scale [40] for the baseline and 1-year follow-up images. This was accomplished by overlaying the ETDRS grid onto the UWF images using Optos software, followed by masking the peripheral fields (See Supplementary Fig. 2). Any discrepancy in grading was resolved with open adjudication among the graders and by discussion with the reading centre director (SRS). A single final result was generated for every case. The same baseline dataset without annotations was transferred to Eyenuk, Inc. for automated haemorrhage detection using the EyeRead UWF software (Fig. 1C, D, E). The EyeRead UWF software is prototype software for research use only and has been specifically designed for use in UWF pseudocolor images. For both the manual and automatically segmented images, the Optos ‘lesion distribution’ tool was employed to quantify the frequency (number), area of retinal haemorrhages (in mm^2^) and average distance (in mm) of all the detected retinal haemorrhages from the optic nerve centre. The lesion metrics were computed within the ETDRS 7 standard field regions and within the five peripheral/extended fields (See Supplementary Fig. 2). The haemorrhage metrics obtained from the two approaches were compared for their ability to predict progression to PDR over one year.

### Statistical analysis

**Fig. 2:**
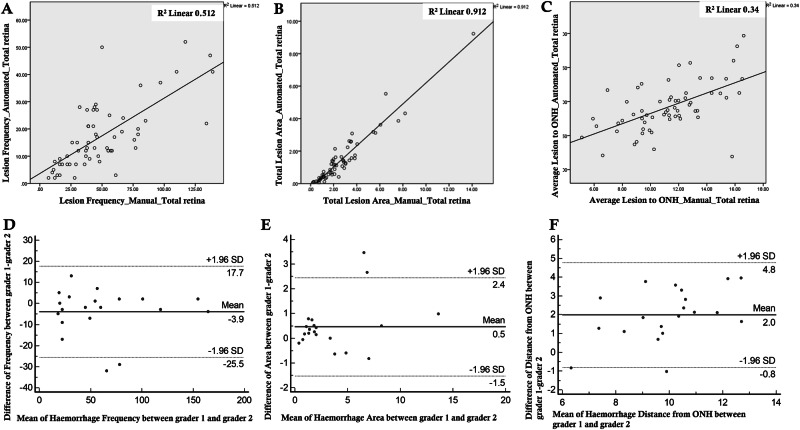
Correlation and reproducibility plots. **A**–**C** illustrate scatter plots depicting the correlation between automatically and manually segmented retinal haemorrhages (2A, total retinal haemorrhage frequency; 2B, total haemorrhage area; and 2C, average distance from the haemorrhage centre to the ONH centre) in the study cohort. Note that the best correlation is depicted in (**B**) (haemorrhage area) with marked clustering of the data points along the line of best fit and least in (**C**) (distance from ONH centre). **D**–**F** show Bland-Altman plots illustrating the reproducibility between the two graders.

The total lesion frequency (N), the total surface area (mm^2^) of retinal haemorrhages and the average distance (mm) of all segmented lesions from the optic nerve and foveal centre were recorded from the output of the Optos ‘lesion distribution’ tool. The Spearman correlation coefficient was used to assess the relationship between the parameters obtained by the two approaches. Scatter plots were generated to illustrate the correlation between the two methods (Fig. 2A–C). Univariable and multivariable regression analysis were performed to compute the odds ratios for each parameter for both approaches. Inter-eye correlations were adjusted using generalised estimating equations during mean difference analysis. Cohen’s kappa coefficient (κ) was used to assess the inter-grader agreement for the manually generated haemorrhage metrics. Bland-Altman plots were constructed to express the difference between the haemorrhage metrics as calculated by grader 1 and grader 2, plotted against their average values. A *P*<0.05 was considered statistically significant.

## Results

A total of 206 eyes were selected at baseline in this retrospective analysis. All eyes with a lack of follow-up data at one year (N = 123), PDR at baseline (N = 11), absence of DR (N = 2), and very poor or ungradable image quality (N = 7) were excluded from the analysis. The poor-quality images were excluded by necessity, since lesion annotation (even by expert graders) on such images can be inaccurate and not reproducible. After excluding these cases, the final study cohort consisted of 63 eyes, among which 29 eyes (46%) progressed to PDR over the 1-year period, whereas 34 eyes did not progress to PDR (See Supplementary Table 1).

The comparison between automated and manual grading is illustrated in Table 1. The total haemorrhage frequency and area as determined using the automated tool, were significantly lower compared to the manual grading. This discrepancy was present when considering only the lesions within the region of the UWF image circumscribed by the EDTRS 7SF and when only considering lesions in the more peripheral regions (the five extended fields). With regards to the average distance of the haemorrhage centre from the ONH centre, manually annotated lesions were on average significantly further from the ONH compared with automatically identified lesions when considering the lesions within ETDRS 7SF alone. In contrast, when considering only the peripheral lesions, the distance to the ONH was comparable for automated and manual approaches.

**Table 1:** Comparative analysis of retinal haemorrhage metrics in manual and automated grading.

	Automated	Manual	*p*
**Within the ETDRS 7 fields**			
Total haemorrhage frequency	12.1 ± 9.21 (1─ 44.19)	38.13 ± 25.96 (4.57─ 104.22)	<0.001
Total haemorrhage area	1.08 ± 1.27 (0.01─ 7.89)	1.97 ± 1.94 (0.1─ 12.78)	<0.001
Average distance to ONH	7.3 ± 1.31 (3.69─ 11.52)	10.09 ± 1.99 (5.54─ 14.61)	<0.001
**Within the peripheral 5 extended fields**			
Total haemorrhage frequency	5.23 ± 4.92 (0─20.22)	10.86 ± 10.69 (0─43.01)	<0.001
Total haemorrhage area	0.37 ± 0.4 (0─1.4)	0.62 ± 0.62 (0─2.9)	<0.001
Average distance to ONH	12.49 ± 2.68 (7.62─ 18.51)	12.56 ± 4.66 (0─ 24.24)	0.25
**Combined (whole retina)**			
Total haemorrhage frequency	17.32 ± 12.16 (2─ 52)	48.99 ± 32.13 (7─ 139)	<0.001
Total haemorrhage area	1.45 ± 1.54 (0.04─ 9.23)	2.58 ± 2.27 (0.15─ 14.11)	<0.001
Average distance to ONH	9.62 ± 1.8 (5.97─ 14.84)	11.32 ± 2.77 (5.13─ 16.61)	<0.001

All the values are in mean ± standard deviation (range), *ETDRS* Early Treatment of Diabetic Retinopathy Study, *ETDRS 5 fields* The central 7 fields (fields 1–7) as defined by the modified Airlie House classification, *Peripheral 5 fields* Peripheral fields corresponding to the central fields (field 3-field 7), present outside the standard ETDRS fields.

Total haemorrhage frequency: in numbers; Total haemorrhage area: in mm^2^; Average distance to ONH: Average distance of the centre of each haemorrhage to the centre of ONH, in mm.

*p* < 0.05 is significant.

Table 2 illustrates the correlation between the automatically annotated and manually annotated lesion metrics. Although correlations were significant for all lesion metrics, the correlation coefficient was highest for the total haemorrhage area and lowest for the average distance to the ONH centre. The same trend was observed for lesions within the EDTRS 7SF, the peripheral five extended fields and all fields combined.

**Table 2:** Correlation between the automated and manually graded retinal haemorrhage metrics.

Automated Vs Manual	r	p
**With the ETDRS 7 fields**		
Total haemorrhage frequency	0.68	<0.001
Total haemorrhage area	0.96	<0.001
Average distance to ONH	0.5	<0.001
**Within the peripheral 5 extended fields**		
Total haemorrhage frequency	0.73	<0.001
Total haemorrhage area	0.88	<0.001
Average distance to ONH	0.6	<0.001
**Combined (whole retina)**		
Total haemorrhage frequency	0.72	<0.001
Total haemorrhage area	0.96	<0.001
Average distance to ONH	0.58	<0.001

*ETDRS* Early Treatment of Diabetic Retinopathy Study, *ETDRS 5 fields*, The central 7 fields (fields 1–7) as defined by the modified Airlie House classification, *Peripheral 5 fields*, Peripheral fields corresponding to the central fields (field 3-field 7), present outside the standard ETDRS fields.

Total haemorrhage frequency: in numbers, Total haemorrhage area: in mm^2^, Average distance to ONH: Average distance of the centre of each haemorrhage to the centre of ONH: in mm, r: Spearman correlation coefficient, p values <0.05 are significant.

Univariable logistic regression analysis showed a strong association between the total number of retinal haemorrhages from the automatic analysis and the distance from the ONH centre from the manually segmented lesions, with progression to PDR. The distance from the ONH centre obtained from both manual (odds ratio OR 0.66; 95% CI 0.44–0.97, *p* = 0.04) and automated (OR 0.64; 95% CI 0.41–0.99, *p* = 0.045) approaches (Table 3) was noted to have a strong association with progression in multivariable analysis.

**Table 3:** Logistic regression analysis to assess the risk of progression to PDR using automated and manual grading.

	Univariate analysis			Multivariate analysis		
**Automated grading**	*Odds Ratio*	95% *CI*	*p*	*Odds Ratio*	95% *CI*	*p*
Total haemorrhage frequency	1.05	1.00–1.10	**0.047**	1.06	0.95–1.18	0.33
Total haemorrhage area	1.16	0.82–1.65	0.39	1.14	0.37–3.47	0.82
Average distance to ONH	1.2	0.89–1.60	0.23	0.64	0.41–0.99	**0.045**
**Manual grading**						
Total haemorrhage frequency	1.01	1.00–1.03	0.13	1.02	0.94–1.05	0.12
Total haemorrhage area	1.08	0.86–1.36	0.5	0.87	0.60–1.27	0.47
Average distance to ONH	1.31	1.07–1.62	**0.01**	0.66	0.44–0.97	**0.04**

Total haemorrhage frequency: in numbers, Total haemorrhage area: in mm^2,^ Average distance to ONH, Average distance of the centre of each haemorrhage to the centre of ONH in mm, 95% CI: 95% confidence intervals.

*p*-values <0.05 are significant and are highlighted in bold.

There was a high level of agreement between the two independent graders for the manual grading of retinal haemorrhages (AV and MGN). The interclass correlation coefficient (95% confidence intervals, CI) and the respective p values for different lesion metrics were as follows: haemorrhage frequency (0.98 [0.96–0.99]; *p* < 0.001); haemorrhage area (0.98 (0.94–0.99); *p* < 0.001; and the average distance from the ONH centre (0.83 (0.57–0.93); *p* < 0.001). Bland-Altman plots were generated to better illustrate the difference between the automated and manual metrics (Fig. 2D–F).

## Discussion

In this study, we observed that a deep-learning system designed for automatically segmenting retinal haemorrhages on UWF pseudocolor images detected a significantly smaller area of haemorrhages compared to a purely manual approach. Although the area of detected lesions was smaller, the automated and manual measurements were significantly correlated and these automated measurements were still predictive of progression to PDR. When considering the average distance of lesions to the ONH centre, the manual and automated approaches showed good agreement when considering only lesions present in the peripheral fields outside the ETDRS 7SF. For lesions present within the ETDRS 7SF, the automated approach yielded a smaller average distance, suggesting that the automated algorithm tended to detect proportionately more lesions in the more central portion of the ETDRS 7SF lesion.

While the explanation for the algorithm’s performance is not certain, we did gain some insight through a post-hoc subjective comparison of the algorithm versus manually segmented lesions. For example, it appeared that the discrepancy in total lesion area was more driven by the determination of the precise borders of the lesion, rather than the presence or absence of a lesion at a given location. Identifying the lesion number only requires the centre or some point within the lesion to be detected. For the lesion area to be computed, every pixel along the border of the lesion must be defined. Thus, total lesion area is a much more difficult task than lesion number. Retinal haemorrhages, especially those in the nerve fibre layer, can have fuzzy and indistinct borders, leading to erroneous determination of the exact border of the lesion [18–20, 22, 25–28, 31, 32]. Since the greatest number of pixels (and hence the greatest contribution to the area measurement) will be along the perimeter of the lesion, small inaccuracies in determining the lesion border can result in large discrepancies in area, especially for larger haemorrhages. Our subjective post-hoc review of automatically detected haemorrhages (Fig. 1) suggests that these errors may have contributed to the absolute discrepancy between manual and automated measurements. This explanation is also consistent with the strong correlation between automated and manual lesion areas (r = 0.96, *p* < 0.001 for central ETDRS fields; r = 0.88, *p* < 0.001 for peripheral fields; and r = 0.96, *p* < 0.001 for combined fields) despite the absolute difference in area.

Given the strong correlation, one might anticipate that automated measurements would still demonstrate an association with DR progression. Indeed, the multivariable regression analysis demonstrated that a greater distance of the haemorrhages from the optic nerve centre was a significant predictor of progression to PDR for both the manual and automated techniques. These results are not surprising, since more peripheral haemorrhages are generally a predictor of greater amounts of peripheral nonperfusion and this has been established to increase the risk of progression to PDR [9, 10, 41]. An important implication of our results is that an AI algorithm for lesion detection does not need to be maximally sensitive in order to be of predictive value.

Our study does have several limitations that must be considered when assessing our results. First and foremost, our analysis is post-hoc and retrospective and thus is subject to ascertainment bias. Overall, two-thirds of initially identified NPDR patients were ultimately excluded as they did not meet our inclusion criteria. We also do not know how the algorithm would perform with lower quality images, since these were excluded by necessity (even manual lesion annotation on lower quality images is not reliable and reproducible, as mentioned earlier). A second limitation and consequence of the many excluded cases is the relatively small sample size. As a result, our study may have been underpowered to identify other parameters that may have contributed to the risk of progression. A third limitation is that we focused on only two metrics, the retinal haemorrhage area and the average distance to the ONH centre. We should note that we focused on these two parameters because these were the two independent predictors for progression in our previous study based on tedious manual segmentation [13]. It is possible, however, that in the setting of an automated algorithm, other DR features (microaneurysms, cotton wool spots, venous beading, IRMA, etc.) could have possibly contributed additional independent risk in our model. These other parameters may be of particular relevance, since the present analysis looked at 1-year progression risk, whereas our previous study looked at risk to progression over four years. Quantifying features such as IRMA and venous beading, however, is challenging and may be a topic of future investigation. It should be noted that in studies that have evaluated peripheral lesions in DR eyes in an effort to identify PPLs, the majority of these lesions were haemorrhages and microaneurysms. Thus, we speculate that when the entire fundus is considered, as is possible with UWF imaging, haemorrhages alone may serve a relatively good surrogate indicator of DR severity. A fourth limitation is that our cohort was exclusively Indian and thus included patients with generally more darkly pigmented fundi. It is well-established that ethnicity and fundus pigmentation can significantly impact AI algorithm performance [42]. Further research will be required to determine how the algorithm performs with other, more diverse cohorts, as our results may not generalise. Our study also has notable strengths, including using the experienced reading centre graders and the stereographic projection to ensure that all lesions, regardless of their location, had accurate physical dimensions for area and distance computation.

In summary, while automated haemorrhage detection on UWF pseudocolor images appears to underestimate the total area of these lesions significantly, the measured area still correlates well with the manual ground truth. Automatically computed average distance between lesions and the ONH centre appears to be associated with a higher risk of progression to PDR. These findings highlight the importance of not only considering the type of lesion and its extent, but also its location, when staging DR. Future studies, including automatic detection of other DR features, may further enhance the predictive model.

## Summary

### What is known about this topic

- Reliable detection of referral-warranted retinopathy by automated algorithms has been demonstrated in several pivotal trials.
- A few studies have also shown that artificial intelligence tools can automatically quantify some diabetic lesions, but have not demonstrated whether these automatically identified lesions can predict retinopathy progression.

### What this study adds

- This study defines the level of agreement between automated and manual methods. It shows that while there is an underdetection by automated methods, the values are still correlated and predictive of progression to proliferative diabetic retinopathy (PDR). The automated approach correlated well with manual lesion detection.
- The distance from the optic nerve emerged as an independent risk of progression with both approaches.
- Although the automated approach yielded lower lesion metrics when compared to the manual, it has the potential to predict the progression to PDR over time.

## Supplementary information

Figure 2

Table 1

Eye reporting checklist

Figure 2 (supplemental)

## Data Availability

The data used for this study is not publicly available to protect participant privacy. However, upon reasonable request, the corresponding author can be contacted at sadda@doheny.org to support reproducibility and transparency as per journal guidelines and policies.
