# Supplementary figures and images for: Predicting progression to proliferative diabetic retinopathy using automated versus manual quantification of retinal haemorrhages
Source: Eye (Lond). 2026 Jan 16;40(5):682–8. doi: 10.1038/s41433-025-04205-2 (PMC13013962; doi:10.1038/s41433-025-04205-2)

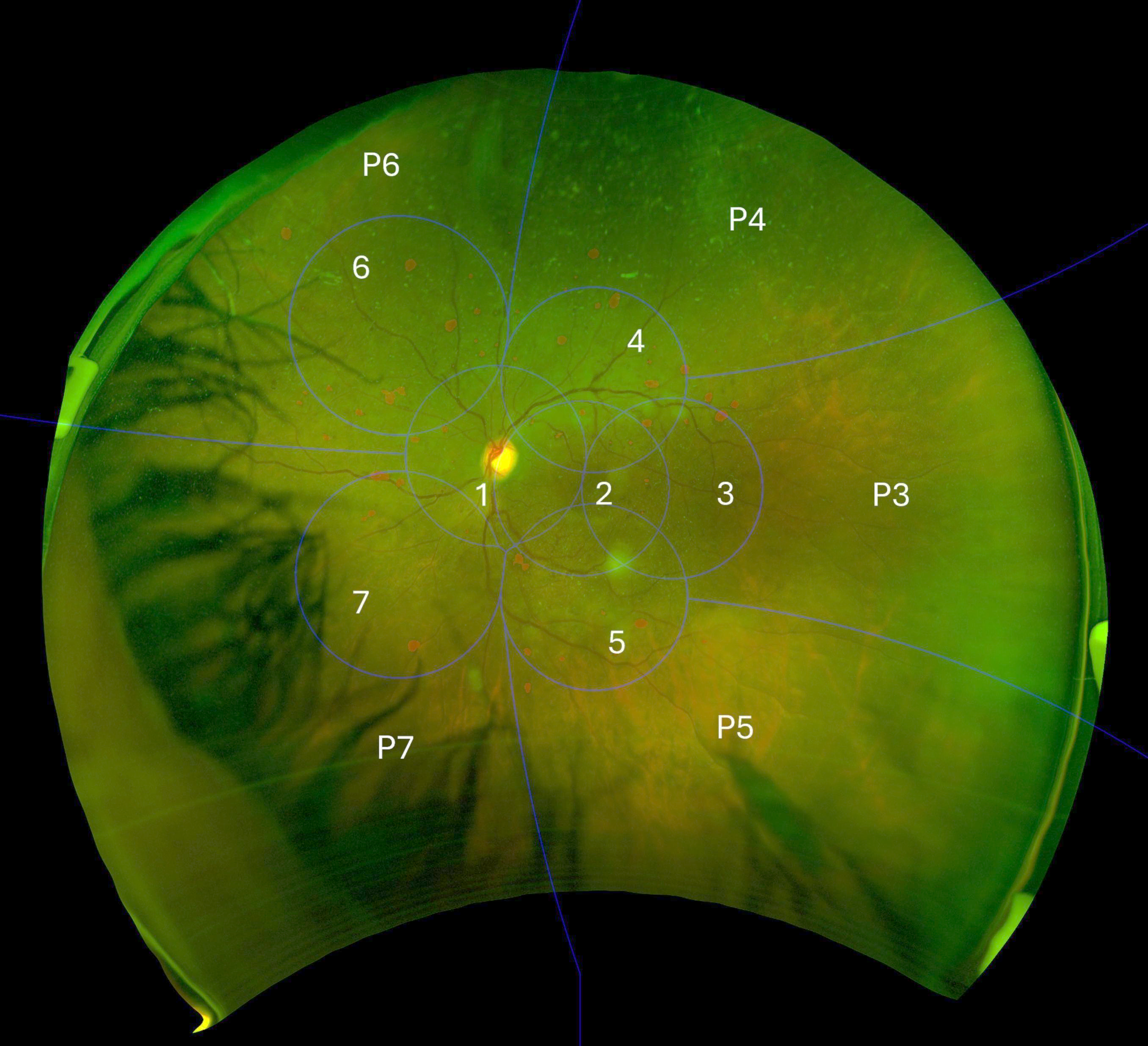

Supplement: Supplementary file 1 — Figure 2 [file 41433_2025_4205_MOESM1_ESM.tif]
