# Supplementary material for: Predicting progression to proliferative diabetic retinopathy using automated versus manual quantification of retinal haemorrhages
Source: Eye (Lond). 2026 Jan 16;40(5):682–8. doi: 10.1038/s41433-025-04205-2 (PMC13013962; doi:10.1038/s41433-025-04205-2)
Supplement: Supplementary file 2 — Table 1 [file 41433_2025_4205_MOESM2_ESM.docx]

**Table 1: Distribution of DR severity grading at baseline and 1-year follow up**

| DR severity at Baseline | Progression to PDR at 1 year follow up (%) |
| --- | --- |
| Moderate NPDR = 45 | 18 (40%) |
| Severe NPDR = 18 | 11 (61.1%) |
| Total = 63 | 29 (46%) |

Abbreviations: DR: Diabetic retinopathy, NPDR: Non-proliferative DR; PDR: Proliferative DR.
