# Supplementary material for: Predicting progression to proliferative diabetic retinopathy using automated versus manual quantification of retinal haemorrhages
Source: Eye (Lond). 2026 Jan 16;40(5):682–8. doi: 10.1038/s41433-025-04205-2 (PMC13013962; doi:10.1038/s41433-025-04205-2)
Supplement: Supplementary file 4 — Figure 2 (supplemental) [file 41433_2025_4205_MOESM4_ESM.docx]

Figure 2 (supplemental): Optos output of an UWF image illustrating the ETDRS grid overlay on the image with segmented retinal haemorrhages. ETDRS seven fields are numbered 1 through 7, whereas the peripheral five extended fields are numbered P3-P7, corresponding to the central field 3- field 7.
